# The Coming of Age of Multicultural Medicine

**DOI:** 10.1371/journal.pmed.0020062

**Published:** 2005-03-29

**Authors:** Gail McBride

## Abstract

Meeting the challenge of providing health care for a multicultural population is now a major movement that is impacting health care worldwide

In Stockton, California, a city of 269,000 people nestled in California's largest agricultural valley, residents are reported to speak 100 different languages. Acculturation is difficult in the best of circumstances, but what happens when those people with limited or no proficiency in English have a medical problem? Many United States hospitals are required to provide some manner of interpreter services for people with limited English proficiency—but do those services also bridge the cultural divide?

Meeting the challenge of providing health care for a multicultural population is now a major movement that is affecting health care in developed countries, principally the US but also in European countries and Australia. Although the bulk of studies and commentaries on the subject began to appear in the 1990s, the literature dates back much further, to articles written in the 1960s and 1970s by medical anthropologists, sociologists, nurses, mental health professionals, and others.

## Wake-Up Calls

In the US, the first major alert on this problem came in 1985, when the *Report of the Secretary's Task Force on Black and Minority Health* was issued [1]. (The “Secretary” was the head of the Department of Health and Human Services (DHHS).) The report painted a bleak picture of the quality of health care afforded to African-Americans and other racial and ethnic minorities.

**Figure 1:**
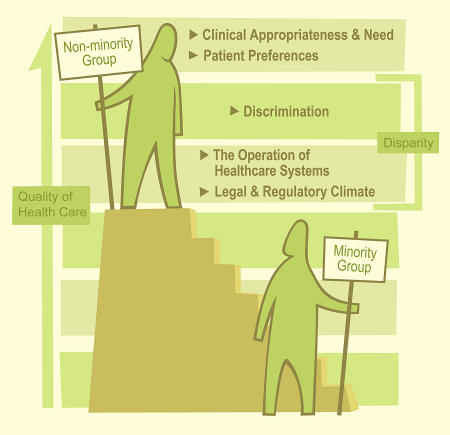
Minority Groups Face a Disparity in the Quality of Health Care (Illustration: Giovanni Maki, adapted from [2]). The Institute of Medicine defined “disparities” in health care as racial or ethnic differences in the quality of health care that are not due to access-related factors, clinical needs, patient preferences, or appropriateness of intervention [2].

A decade later, reports from the US Institute of Medicine began to appear. Three of the ten reports, which spanned a ten-year period, dealt with the need to greatly diversify the health professions work force—still a somewhat unachieved goal. The most recent, considered a new wakeup call, was the 2003 report *Unequal Treatment: Confronting Racial and Ethnic Disparities in Health Care* [2]. It minced few words in describing the problems faced by racial and ethnic minorities who sought health care: “The conditions in which many clinical encounters take place—characterized by high time pressure, cognitive complexity, and pressures for cost-containment—may enhance the likelihood that these processes will result in care poorly matched to minority patients' needs. Minorities may experience a range of other barriers to accessing care, even when insured at the same level as whites, including barriers of language, geography and cultural familiarity” (Figure 1).

Soon afterward, another US government arm, the Agency for Healthcare Research and Quality of the DHHS, issued two other reports: the *National Healthcare Disparities Report* [3] and the *National Healthcare Quality Report* [4], with annual updates promised. The reports focused on seven clinical conditions, including cancer, diabetes, and mental health, and discussed the quality of care and differences in access to such care for special population groups, including minorities and the disabled.

All of these reports make it clear that health care professionals and health systems need to change. In recent years, in order to improve their lives economically or avoid war and/or famine, many people have migrated from less to more developed areas of the world, changing the demographics of the US and a number of other societies. Evidence that they and nonmigrant minorities experience inequities in attaining quality health care is abundant [5].

Studies also indicate that although genetics is involved in some health-related differences between racial and ethnic groups, such as in the incidence of certain diseases and responses to pharmaceuticals, it is probably not a major factor in explaining health disparities [6].

## The Era of Action

A primary result of these reports on health disparities? A truly dizzying array of offices, centers, programs, and initiatives within the main DHHS as well as in some of its major branches such as the National Institutes of Health and the Centers for Disease Control and Prevention, all designed to improve health care for racial and ethnic minorities in one way or another. Some of these programs also fund grants to outside organizations, public and private, and coordinate with state offices of minority health.

And there are more activities devoted to reducing health disparities: (1) university-level institutes, offices, and programs, such as those at the UMDNJ-Robert Wood Johnson Medical School and Georgetown University, (2) private foundations, such as the California Endowment, (3) agencies and programs within the various states, such as the very active Ohio Commission on Minority Health, and (4) combinations of groups, such as DiversityRx (www.diversityrx.org), an informational organization sponsored by the National Conference of State Legislatures, Resources for Cross-Cultural Health Care, and the Henry J. Kaiser Family Foundation.

All these efforts might suggest that there are no problems left to be solved, but this is hardly the case. Providing quality health care to those who differ from a country's majority population in terms of language and culture (and often race) is a mammoth task that does not yield to easy or quick fixes, but rather to consistent and determined efforts at improvement.

## Cultural Competence

The most common term used in this effort is “cultural competence,” essentially defined as a respectful knowledge of and attitude toward people from different cultures that enables health professionals who work with people from another culture to develop and use standard policies and practices that will increase the quality and outcome of their health care.

With cultural competence as the centerpiece, social and behavioral scientists have started consulting companies to (1) train health care professionals working in private and public health care settings (hospitals, community clinics, managed health care plans) in cultural competence, and (2) propose as well as study the effects of such changes in these settings. Some hospitals and managed health care plans have developed their own programs; examples that stand out are the M.D. Anderson Hospital in Texas and Kaiser Permanente health plans.

In 2000, the M.D. Anderson Hospital established an Office of Institutional Diversity, which emphasizes the use of employees with a variety of backgrounds and experiences to examine cancer and its impact on all kinds of people. Educational forums, employee network groups, and the use of evidence-based hypotheses to design and implement pilot interventions are all part of the effort to improve care of culturally diverse patients.

**Figure:**
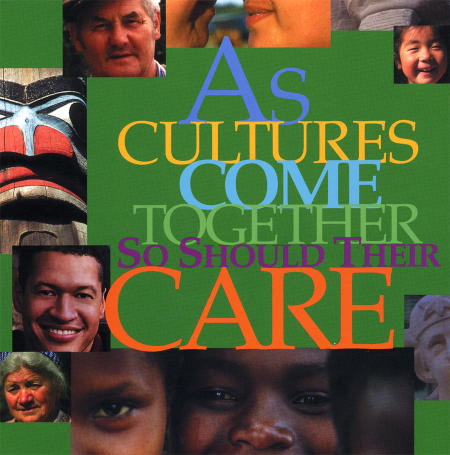


Kaiser Permanente's Institute for Culturally Competent Care selects and coordinates Kaiser Permanente's several Centers of Excellence, which each serve specific populations. For example, a West Los Angeles center focuses on the diagnosis, treatment, and management of conditions prevalent among African-Americans, such as sickle cell disease and prostate cancer. The National Diversity Department emphasizes a diverse workforce and has published a number of providers' handbooks on culturally competent care for specific racial or cultural patient groups, such as Latino patients.

## Not to Be Left Out

Pharmaceutical companies have also discovered multicultural medicine. Many that offer continuing medical education courses to help publicize their new drugs now also offer courses on diseases more prevalent in certain racial and ethnic groups than others (such as diabetes in the Hispanic/ Latino population). These courses include information on how to treat such groups with the company's drugs.

Interestingly, in 2004 a clinical trial proved the effectiveness of the first drug specifically designed for the treatment of congestive heart failure in African-Americans [7]. The drug, a combination of fixed doses of isosorbide and hydralazine, may now be nearing the market. Despite the fact that the Association of Black Cardiologists was a cosponsor of the trial, the trial drew criticism on the basis that it allowed race to interfere with treatment decisions [6].

## A Global Issue

The increased diversity of European populations, with the expected stress on entrenched health care systems and on the migrants themselves, has led to Migrant-Friendly Hospitals (http://www.mfh-eu.net), a “European initiative to promote health and health literacy of migrants and ethnic minorities” begun in October 2002.

With funding from the European Commission and the Austrian Federal Ministry for Education, Science and Culture, a network of 12 pilot hospitals from European Union member states has been implementing and evaluating the effectiveness of three health care models for migrants and minorities. The models are: the improvement of interpreting in clinical communication, the creation and distribution of migrant-friendly information and training in mother and child care, and staff training in cultural competence. Results of the pilot experiences were reported at a final conference in December 2004 and will form the basis of European recommendations on migrant-friendliness as a quality criterion for hospital development and on the role of hospitals in promoting health and health literacy for migrants and ethnic minorities.

One of the 12 pilot hospitals mentioned above is the Bradford Hospitals NHS Trust, long active among a number of other hospitals and health projects in the UK that strive to improve services for racial and ethnic minorities in their areas.

Australia also has a multicultural society, and The Centre for Culture and Health of the University of New South Wales in Sydney has an active program aimed at increasing cultural competency, both among medical students at the University and in the country's medical community at large (http://cch.med.unsw.edu.au/). The Centre offers graduate certificates and diplomas in public health (culture and health), as well as a Masters in Public Health with a concentration in multicultural health, and a postgraduate research degree. It emphasizes the establishment of partnerships with Area Health Services around New South Wales, grassroots organizations, and governmental organizations. A number of research projects also are underway. There are, for example, intervention strategies designed to reduce risk for cardiovascular disease in various cultural groups, such as the Arabic and Farsi-speaking communities, and studies of cancer among Chinese families in Australia.

## Conclusion

People's basic medical needs do not vary greatly; they can be accommodated with appropriate understanding, awareness, and education. In the end, medicine and health care can only be enhanced and informed by the broadening of cultural awareness.

**Further Reading on Multicultural Medicine**

Here are three captivating books that yield knowledge through narrative.

- *The Spirit Catches You and You Fall Down: A Hmong Child, Her American Doctors, and the Collision of Two Cultures* by Anne Fadiman (Farrar, Straus and Giroux, 1997).
- *Healing by Heart: Clinical and Ethical Stories of Hmong Families and Western Providers* by Kathleen Culhane-Pera and coauthors (Vanderbilt University Press, 2003).
- *Healing Latinos: Realidad y Fantasia*, a collection of physician-patient vignettes edited by David E. Hayes-Bautista and the late Roberto Chiprut (Cedars Sinai Health Systems and the UCLA Center for Latino Health, 1998).

## Abbreviation

DHHS: Department of Health and Human Services
